# Sensitive determination of the spin polarization of optically pumped alkali-metal atoms using near-resonant light

**DOI:** 10.1038/srep32605

**Published:** 2016-09-06

**Authors:** Zhichao Ding, Xingwu Long, Jie Yuan, Zhenfang Fan, Hui Luo

**Affiliations:** 1 College of Optoelectronic Science and Engineering, National University of Defense Technology, Changsha 410073, China; 2 Interdisciplinary Center for Quantum Information, National University of Defense Technology, Changsha 410073, China

## Abstract

A new method to measure the spin polarization of optically pumped alkali-metal atoms is demonstrated. Unlike the conventional method using far-detuned probe light, the near-resonant light with two specific frequencies was chosen. Because the Faraday rotation angle of this approach can be two orders of magnitude greater than that with the conventional method, this approach is more sensitive to the spin polarization. Based on the results of the experimental scheme, the spin polarization measurements are found to be in good agreement with the theoretical predictions, thereby demonstrating the feasibility of this approach.

Since the ingenious idea of optical pumping was proposed by Kastler in 1950^1^, it has played an important role in atomic physics^2^,^3^,^4^. Optical pumping is typically used to polarize alkali-metal atoms. Once an ensemble of alkali metal atoms is polarized, many perfect physical properties can be observed^2^. As a result, optically pumped alkali-metal vapor has been widely used in a variety of significant areas, such as atomic magnetometers^5^,^6^,^7^,^8^,^9^, Faraday filters^10^,^11^,^12^, atomic clocks^13^, quantum memory and teleportation^14^,^15^, and nuclear magnetic resonance^16^,^17^. The spin polarization, which reflects the spin coherence of an atomic ensemble, is a vital parameter of optically pumped alkali-metal atoms. For example, the spin polarization directly determines the performance of atomic magnetometers and has an optimal value for an atomic magnetometer^6^,^8^, and it is also helpful for people to research and design Faraday filters by obtaining accurate knowledge of the spin polarization^12^. Therefore, it is essential to measure the spin polarization of alkali-metal atoms accurately.

The spin polarization is usually determined by Faraday rotation using far-detuned light^18^,^19^. In such a measurement, for the D_1_ line transition of alkali-metal atoms, the Faraday rotation angle *θ* is given by^19^


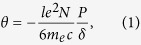


where *l* is the length over which the probe light interacts with the alkali-metal vapor, *e* is the electron charge, *N* is the number density of the alkali-metal vapor, *m*_*e*_ is the electron mass, *c* is the speed of light, *δ* is the probe detuning from the D_1_ line transition, and *P* is the spin polarization of the alkali-metal atoms. A necessary condition of equation (1) is that *δ* is much greater than the hyperfine splitting^19^. In this case, *θ* is scarcely sensitive to *P*, unless *N* is large enough. For the vapor number density of 10^11^ to 10^12^ cm^−3^, as *θ* is usually only several milliradians^18^, it is difficult to obtain accurate knowledge of the spin polarization using this method.

However, in many practical applications, alkali-metal vapor operating at near room temperature, which corresponds to the number density close to 10^11^ cm^−3^, has great advantages and has been widely utilized. For example, room temperature operation can simplify the structure and can reduce the energy consumption for atomic magnetometers, as indicated by the many reported room temperature atomic magnetometers^8^,^20^. Therefore, a sensitive method to measure the spin polarization is of great value, especially for the vapor with low number density.

In this paper, a new approach to determine the spin polarization of alkali-metal atoms is proposed. Instead of far-detuned probe light, near-resonant light is used. By comparing the detected Faraday rotation angles of the probe light at two specific frequencies, the difference is nearly proportional to the spin polarization. As the Faraday rotation angle using near-resonant probe light can be two orders of magnitude larger than that using far-detuned light, this approach is much more sensitive to the spin polarization. Therefore, in terms of the alkali-metal vapor with low number density, the proposed method is more suitable than the traditional method to determine the spin polarization accurately. In addition, the Faraday rotation is also utilized in many types of atomic magnetometers, such as the classical *M*_*x*_ magnetometer and magnetometer based on nonlinear magneto-optical rotation^21^,^22^. Thus, the detection method of this paper can be used in such magnetometers, and it may be a valid means to improve the sensitivity of atomic magnetometers. To theoretically illustrate and experimentally verify this method, the ^87^Rb atom is considered.

## Methods

### Theoretical Background

The ^87^Rb atom is able to absorb photons at frequencies other than the resonance frequency. Because of natural broadening and pressure broadening and shift, the lineshape of the atomic frequency response around the resonance frequency *ν*_0_ to the light of frequency *ν* has the form of a Lorentzian curve *L(ν* − *ν*_0_). In addition, because of Doppler broadening, the lineshape has the form of a Gaussian curve *G(ν* − *ν*_0_). Synthesizing these effects, the resulting lineshape is the Voigt profile *V(ν* − *ν*_0_), which satisfies^23^


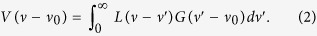


For the ^87^Rb D_1_ transition *F* → *F*′, considering the contributions of each hyperfine transition, the absorption cross-section *σ(ν*) is^23^


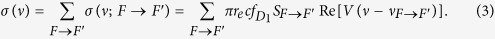


Here, *F* and *F*′ represent the total angular momentum of the ^87^Rb atom in the ground state and the first excited state, respectively. *r*_*e*_ is the classical electron radius, *f*_*D*1_ is the oscillator strength of the D_1_ line, *ν*_*F*→*F*′_ is the resonance frequency of the transition *F* → *F*′, and Re[*V(ν* − *ν*_*F*→*F*′_)] is the real part of *V(ν* − *ν*_*F*→*F*′_). *S*_*F*→*F*′_ is the relative transition strength, which is given by^24^.


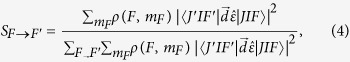


where *m*_*F*_ = −*F*, −*F* + 1, …, *F* is the magnetic quantum number of the ^87^Rb atom. *ρ(F*,*m*_*F*_) is the probability of the ^87^Rb atom being in state 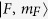. As nearly all the ^87^Rb atoms are in the ground state at any time in the scope of our discussion, 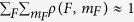. *J* and *J*′ represent the electron angular momentum of the ^87^Rb atom in the ground state and the first excited state, respectively. *I* represents the nuclear spin, 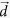 is the dipole-moment vector, and 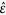 is the unit vector of light polarization. Expanding 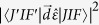 in the spherical-basis, one easily obtains the following^24^.


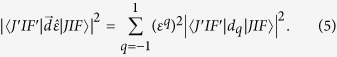


Here, *d*_*q*_ and *ε*^*q*^ are the *q*-*th* spherical components of the dipole-moment vector and the unit vector of light polarization, respectively. For left-circularly polarized light propagating along the z axis, all spherical components are zero except *ε*^1^ = 1. Similarly, for right-circularly polarized light propagating along the z axis, all spherical components are zero except *ε*^−1^ = 1. Using the Wigner-Eckart theorem^24^, we can obtain


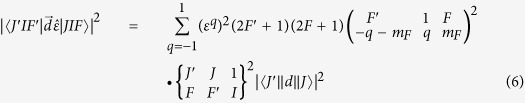


Because linearly polarized light can be considered as the superposition of left-circularly and right-circularly polarized light, for linearly polarized light propagating along the z axis, *S*_*F*→*F*′_ = *S*_*F*→*F*′,+_ + *S*_*F*→*F*′,−_, where *S*_*F*→*F*′,+_ and *S*_*F*→*F*′,−_ are the relative transition strengths for the left-circularly polarized component and the right-circularly polarized component, respectively, which are given by


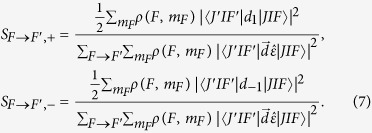


According to equation (6), it is easy to calculate *S*_*F*→*F*′,+_ and *S*_*F*→*F*′,−_. The results are shown in Table 1.

When an ensemble of ^87^Rb atoms in a spherical cell is in the equilibrium state, the difference in the number of atoms between different Zeeman energy levels is tiny. In this situation, if linearly polarized light propagating along the z axis is used to probe this ensemble and it is too weak to disturb the distribution of ^87^Rb atoms in the ground state, then


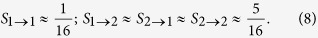


After the probe light passes through the cell, its intensity *I*_*out*_ is described by^23^


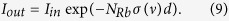


Here, *I*_*in*_ is the intensity of the probe light before entering into the cell, *N*_*Rb*_ is the ^87^Rb vapor density, and *d* is the diameter of the cell. By measuring *I*_*out*_/*I*_*in*_ and then fitting equation (9), *σ(ν*) and *N*_*Rb*_ can be obtained.

To prepare the spin polarization, assume that left-circularly polarized light propagating along the z axis is used to polarize this ^87^Rb ensemble, and a constant magnetic field *B*_*0*_ is applied along the z axis to achieve effective optical pumping^2^. The spin polarization of ^87^Rb atoms along the z axis *P*_*z*_ is given by


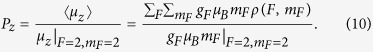


Here, 〈*μ*_*z*_〉 is the expectation value of *μ*_*z*_, which is the magnetic moment of ^87^Rb atoms along the z axis. *g*_*F*_ is the Landé g factor of the ^87^Rb atom. For *F* = 1 and 2, *g*_*F*_ = −1/2 and 1/2, respectively^25^. *μ*_*B*_ is the Bohr magneton. Substituting the values of these parameters into equation (10), we obtain


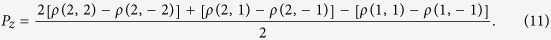


Assume that *ρ*(1, 1) − *ρ*(1, −1) = −2*kP*_*z*_; then, 2[*ρ*(2, 2) − *ρ*(2, −2)] + [*ρ*(2, 1) − *ρ*(2, −1)] = 2(1 − *k*)*P*_*z*_, where *k* is a constant number that depends on the distribution of ^87^Rb atoms in the ground state.

After the linearly polarized probe light passes through the cell, the polarization plane of light will rotate. When the probe light is so weak that we can neglect the nonlinear magneto-optical effects caused by the light field, the rotation angle is given by^26^


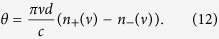


Here, *n*_+_ (*ν*) and *n*_−_ (*ν*) represent the refractive indices for the left-circularly polarized component and the right-circularly polarized component of linearly polarized light, respectively, which are related to the electric susceptibility of the ^87^Rb vapor and given as follows^23^,^27^


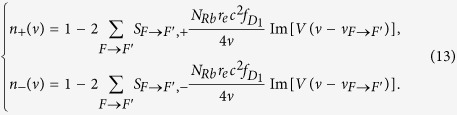


Im[*V(ν* − *ν*_*F*→*F*′_)] is the imaginary part of *V(ν* − *ν*_*F*→*F*′_). Substituting equation (11), equation (13) and Table 1 into equation (12), one can easily obtain


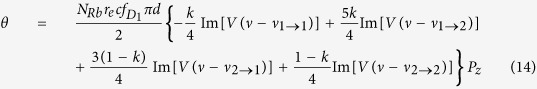


If the condition of


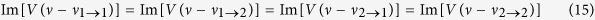


is satisfied for a specific frequency *ν*_*s*_, then *θ* is independent of *k* and can be given by


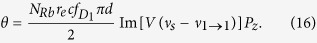


**Figure 1:**
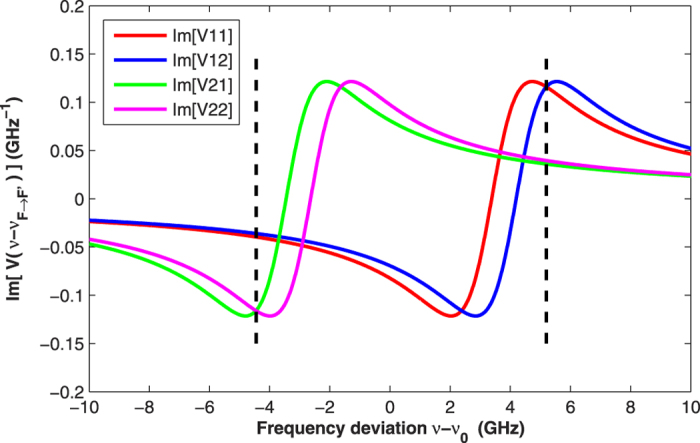
Simulation results of Im[*V(ν* − *v*_*F*→*F*_′)] for each hyperfine transition. Im[*V(ν* − *v*_*F*→*F*_′)] is simplified as Im[*VFF*′]. The simulation conditions are as follows: pressure broadening *γ*_*v*_ = 2.5 GHz, pressure shift *δ*_*ν*_ = −0.4 GHz, and temperature *T* = 343 K.

Thus, we can determine *P*_*z*_ by measuring *θ* with proper *ν*. Figure 1 shows the simulation results of Im[*V(ν* − *ν*_*F*→*F*′_)] for each hyperfine transition as a function of frequency deviation. The simulation conditions are as follows: pressure broadening *γ*_*v*_ = 2.5 GHz, pressure shift *δ*_*ν*_ = −0.4 GHz, and temperature *T* = 343 K (which corresponds to the Doppler broadening of 0.54 GHz)^23^.

Figure 1 clearly shows that the condition of equation (15) is satisfied only when the probe light is far-detuned. Therefore, for the conventional approach, far-detuned light is chosen to measure the spin polarization, even though Im[*V(ν* − *ν*_*F*→*F*′_)] is quite small. However, we find that if two specific frequencies *ν*_*s*1_ and *ν*_*s*2_, which are the right intersection of Im[*V(ν* − *v*_1→1_)] and Im[*V(ν* − *v*_1→2_)] and the left intersection of Im[*V(ν* − *v*_2→1_)] and Im[*V(ν* − *v*_2→2_)], respectively, shown with two black dashed lines in Fig. 1, are selected, then the difference value *θ*_1_ − *θ*_2_ of the detected Faraday rotation angles is nearly proportional to the spin polarization. We can consider that *θ*_1_ − *θ*_2_ is proportional to the spin polarization, although there is a small error, and it is given by


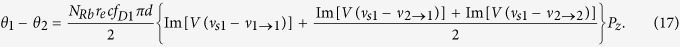


The error of this measuring method comes from the minor difference between Im[*V(ν*_s1_ − *v*_2→1_)] and Im[*V(ν*_s1_ − *v*_2→2_)]. By analyzing the simulation results with different values of *γ*_*v*_, *δ*_*ν*_ and *T*, we assess that this method is valid with an error of less than 2%. Because the Faraday rotation angle of this approach can be two orders of magnitude larger than that of the conventional method, compared with the conventional approach, this method is much more sensitive to the spin polarization.

### Experimental Implementation

**Figure 2:**
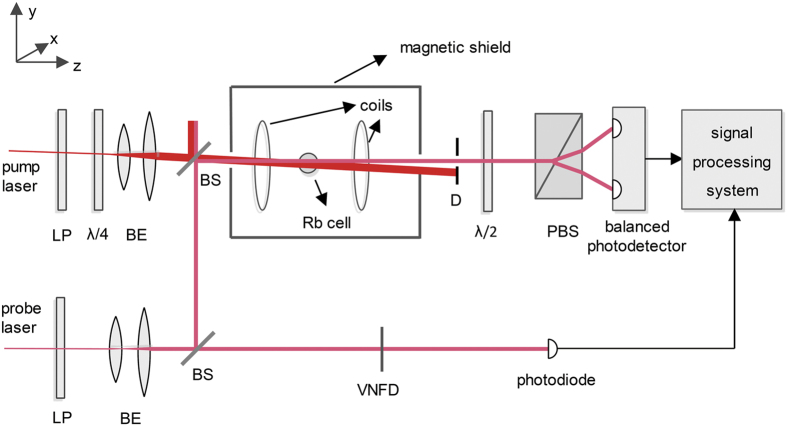
Schematic diagram of the experimental setup. LP: linear polarizer, λ/4: quarter-wave plate, BE: beam expander, BS: beam splitter, D: diaphragm, λ/2: half-wave plate, PBS: polarized beam splitter, VNDF: variable neutral density filter.

The experimental setup is shown in Fig. 2. A spherical Pyrex cell with a diameter of 20 mm, which is also used to study atomic magnetometers, is chosen. The cell contains isotopically pure ^87^Rb atoms and 100 Torr of buffer gas (70 Torr N_2_ and 30 Torr ^4^He) for slowing atomic diffusion and quenching. To avoid the disturbance to the polarized ^87^Rb atoms by the geomagnetic field and disruptive magnetic fields produced by the heaters, the cell is placed inside a five-layer magnetic shield and heated by two sheets of Minco thermofoil. A constant magnetic field *B*_0_ = 11.3 μT along the z axis is generated by the surrounding pair of Helmholtz coils, which is driven by a steady current circuit. The frequency of the probe light is modulated by scanning the current of the laser tube and is measured using a HighFinesse WSU wavelength meter.

The pump beam is generated by a distributed feedback diode laser, which is locked to the resonance frequency of the D_1_ transition *F* = 2 → *F*′ = 1. The pump beam becomes left circularly polarized light after passing through a linear polarizer and a λ/4 plate. After expansion and collimation, the pump beam reaches a 50/50 beam splitter. The transmitted part of the pump beam polarizes the ^87^Rb atoms nearly along the z axis. The residual pump beam is blocked by a diaphragm.

The probe beam is also generated by a distributed feedback diode laser. After passing through a linear polarizer and a beam expander, the probe light reaches a 50/50 beam splitter. The transmitted part of the probe light acts a reference beam and is detected by a photodiode after passing through a variable neutral density filter. After being reflected by another beam splitter, the reflected part of the probe beam illuminates the cell. The intensity and polarization plane of the probe light are detected using a setup involving a λ/2 plate, a polarized beam splitter, and a balanced photodetector. Finally, the detected signals from the photodiode and the balanced photodetector are acquired and processed by a signal processing system.

At room temperature, the total intensity *I*_*tot*_ of the probe light that reaches the two photosurfaces of the balanced photodetector is adjusted to approximately 10 μW/cm^2^. Because the intensity of the probe light will change when the current of the laser tube is scanned, a reference path in the bottom of Fig. 2 is built. The variable neutral density filter is adjusted to set the intensity *I*_*pd*_ of light reaching the photodiode to be equal to *I*_*tot*_. As the absorption of the probe light by ^87^Rb atoms is quite small and can be ignored at room temperature, *I*_*out*_/*I*_*in*_ is equal to *I*_*tot*_/*I*_*pd*_ for any operational temperature.

## Results and Discussion

**Figure 3:**
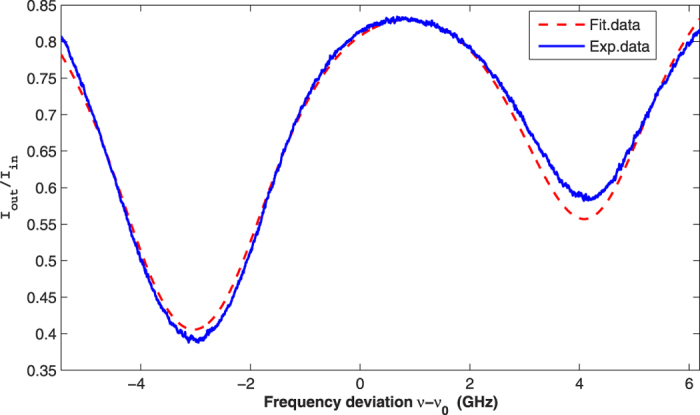
Experimental data (blue solid line) and the fitting data (red dashed line) of *I*_*out*_/*I*_*in*_ as a function of the frequency deviation of the probe light at 338 K.

First, the pump light is shutoff and the constant magnetic field is not applied. Next, the cell is heated to a specific temperature. When the frequency of the probe light is scanned, *I*_*tot*_ and *I*_*pd*_ are recorded simultaneously. The blue solid line in Fig. 3 is the experimental data of *I*_*out*_/*I*_*in*_ as a function of the frequency deviation at 338 K.

The strategy for fitting *I*_*out*_/*I*_*in*_ is as follows. The natural broadening is evaluated by the lifetime of the first excited state. As the Doppler broadening varies with *T* slowly (when *T* varies by 10 K, the change in the Doppler broadening is slightly smaller than 0.01 GHz.), it is calculated using the detected temperature^23^. *δ*_*ν*_ is determined by the left valley of the solid blue line in Fig. 3. By fitting *I*_*out*_/*I*_*in*_ for different *γ*_*v*_ and *N*_*Rb*_ using equations (3), (8) and (9), the best fitting result is obtained, as shown by the red dashed line in Fig. 3.

Figure 3 indicates that the experimental data and the fitting data are nearly identical. Nevertheless, some deviations are present, especially in the two valleys of Fig. 3, and the deviations also exist at other operational temperatures. The main reasons for the deviations are system noise and the slight influence of the probe light on the distribution of the ^87^Rb atoms in the ground state. The system noise, which is caused by the fluctuations of some of the related parameters, will lead to detection errors of *I*_*out*_/*I*_*in*_. The distribution of ^87^Rb atoms in the ground state determines the form of *σ(ν*). When the frequency of the probe light is at the two valleys, *σ(ν*) is relatively large in the scanning scope. As a result, the fluctuations of the frequency and intensity of the probe light and the operational temperature will lead to a relatively high detection error, and the influence of the probe light on the distribution of ^87^Rb atoms in the ground state is relatively strong.

Using the same strategy, we measure and fit *I*_*out*_/*I*_*in*_ under different operational temperatures; the results are shown in Table 2. The right-most column in Table 2 represents the theoretical values of *N*_*Rb*_, which are estimated from the empirical formula of the saturated vapor density^25^. As two copper sheets, on which we placed the temperature sensor, are attached to the cell and two sheets of Minco thermofoil are attached to the two copper sheets, the detected temperature of the ^87^Rb vapor is higher than the actual temperature. Derived from the experimental and theoretical results of *N*_*Rb*_, the difference between the detected temperature and the actual temperature is approximately 5 K for our experimental system. After *γ*_*v*_ and *δ*_*ν*_ are obtained, we can determine Im[*V(ν* − *v*_*F*→*F*_′)], *ν*_*s*1_ and *ν*_*s*2_ as in Fig. 1.

**Figure 4:**
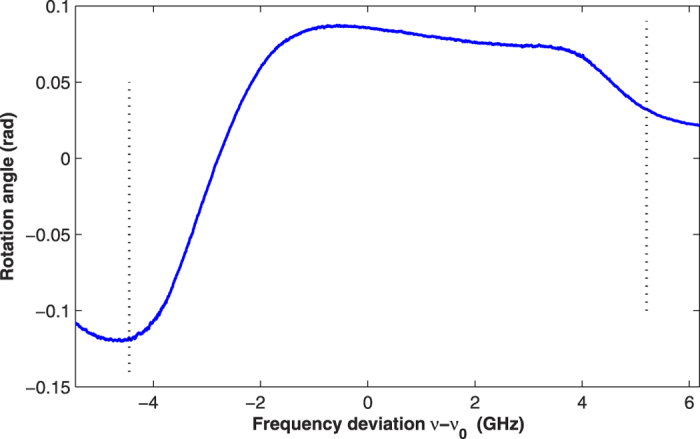
Rotation angle of the probe light as a function of the frequency deviation. The two black dashed lines represent the two specific frequencies, *T* = 338 K, and the intensity of the pump light is 1 mW/cm^2^.

For effectively preparing the spin polarization of the ^87^Rb atoms, the pump beam is utilized when the constant magnetic field along the z axis is applied. Next, the frequency of the probe light is scanned, and the rotation angle of the polarization plane is detected by the balanced photodetector. Figure 4 shows the experimental result at 338 K for the intensity of pump light of 1 mW/cm^2^. The two black dashed lines represent *ν*_*s*1_ and *ν*_*s*2_. The spin polarization is easy to obtain from equation (17).

**Figure 5:**
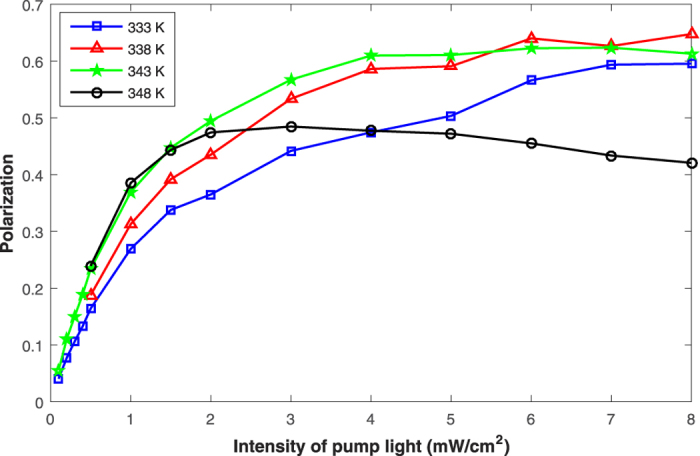
Experimental results of the spin polarization under different temperatures and intensities of the pump light.

By changing the intensity *I*_*pl*_ of the pump light under different operational temperatures, the spin polarization dependence on *I*_*pl*_ is obtained; the results are shown in Fig. 5.

In theory, the spin polarization is equal to the ratio of the optical pumping rate *R*_*op*_ to the total relaxation rate *R*_*tot*_ of the ^87^Rb atoms. As the relaxation rate *R*_*rt*_ due to radiation trapping is positively related to *R*_*op*_^28^, when *I*_*pl*_ is small enough, *R*_*tot*_ is dominated by the relaxation rate due to other relaxation mechanisms that are independent of *I*_*pl*_, and the spin polarization is nearly proportional to *I*_*pl*_. However, with increasing *I*_*pl*_, *R*_*rt*_ gradually becomes the dominant factor of *R*_*tot*_, leading to the spin polarization tending to be stable or decreasing slowly.

With the increase of *T*, the pressure of the buffer gas in the cell increases, leading to the decrease of the relaxation rate due to wall collisions and transit relaxation^24^,^29^. As a result, the spin polarization may increase with *T* when *I*_*pl*_ is small enough. However, with increasing *I*_*pl*_, because *R*_*rt*_ is positively related to *N*_*Rb*_^28^ and it becomes the dominant factor of *R*_*tot*_ gradually, *R*_*tot*_ will increase with *T* when *I*_*pl*_ is large enough. Thus, compared with the case of low operational temperature, the slope of the spin polarization as a function of *I*_*pl*_ will decrease faster under high operational temperature.

The experimental results in Fig. 5 are in good agreement with the theoretical predictions, demonstrating the feasibility of the proposed measurement method for the spin polarization.

## Conclusions

In summary, we demonstrated a sensitive approach to measure the spin polarization of optically pumped alkali-metal atoms. Differing from the conventional method, which uses far-detuned probe light, near-resonant light is selected. It was theoretically proved that the difference in the detected Faraday rotation angles of probe light with two specific frequencies is nearly proportional to the spin polarization, and the error is less than 2%. The measurement results of the spin polarization are found to be in good agreement with the theoretical predictions, demonstrating the feasibility of this measuring method. Because the Faraday rotation angle of this approach can be two orders of magnitude greater than that of the conventional method, the proposed method is much more sensitive to the spin polarization. For an alkali-metal vapor with low number density, this approach is much more suitable for determining the spin polarization accurately.

In addition, the Faraday rotation is also utilized in many types of atomic magnetometers. Thus, the detection method of this paper can be used in such magnetometers. The use of the proposed method may be a valid means to improve the sensitivity of atomic magnetometers; however, further research is required to demonstrate this potential application.

## Additional Information

**How to cite this article**: Ding, Z. *et al.* Sensitive determination of the spin polarization of optically pumped alkali-metal atoms using near-resonant light. *Sci. Rep.* **6**, 32605; doi: 10.1038/srep32605 (2016).

## Figures and Tables

**Table 1:** The relative transition strengths for the left-circularly polarized component *S*_*F*→*F*′,+_ and the right-circularly polarized component *S*_*F*→*F*′,−_ of linearly polarized light.

*F* → *F*'	*S*_*F*→*F*′,+_	*S*_*F*→*F*′,−_
1 → 1	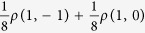	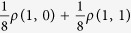
1 → 2	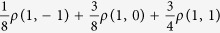	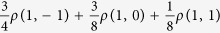
2 → 1	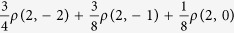	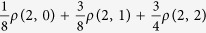
2 → 2	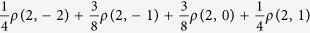	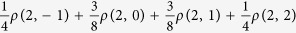

**Table 2:** Optimum parameters for fitting of *I*_*out*_/*I*_*in*_.

Temperature (K)	*γ*_*ν*_ (GHz)	*δ*_*ν*_ (GHz)	*N*_*Rb*_ (10^11^/cm^3^) (in theory)
333	2.52	−0.42	2.39 (3.38)
338	2.58	−0.38	3.53 (5.03)
343	2.63	−0.40	5.06 (7.40)
348	2.69	−0.41	7.18 (10.76)
